# Lack of Processing of the Expressed ORF1 Gene Product of Hepatitis E Virus

**DOI:** 10.1186/1743-422X-8-245

**Published:** 2011-05-20

**Authors:** Suganthi Suppiah, Yumei Zhou, Teryl K Frey

**Affiliations:** 1 Department of Biology, Georgia State University, Atlanta, GA, USA

## Abstract

**Background:**

Proteolytic processing is a common mechanism among plus strand RNA viruses and the replicases of all plus strand RNA viruses of animals thus far characterized undergo such processing. The replicase proteins of hepatitis E virus (HEV) are encoded by ORF1. A previous report published by our group [1] provided data that processing potentially occurred when ORF1 (Burma strain; genotype 1) was expressed using a vaccinia virus-based expression system.

**Findings:**

To further test for processing and to rule out artifacts associated with the expression system, ORF1 was re-expressed using a plasmid-based expression vector with the result that the previous processing profile could not be confirmed. When ORF1 from an HEV infectious cDNA clone (US swine strain; genotype 3) was expressed using the plasmid-based system, the only species detected was the 185 kDa precursor of ORF1. A putative papain-like cysteine protease [2] had been predicted within ORF1 using the original HEV genomic sequence. However, analysis of subsequent ORF1 sequences from a large number of HEV isolates reveals that this protease motif is not conserved.

**Conclusions:**

The expressed HEV ORF1 gene product does not undergo proteolytic processing, indicating that the replicase precursor of HEV is potentially unique in this regard.

## Findings

**Figure 1:**
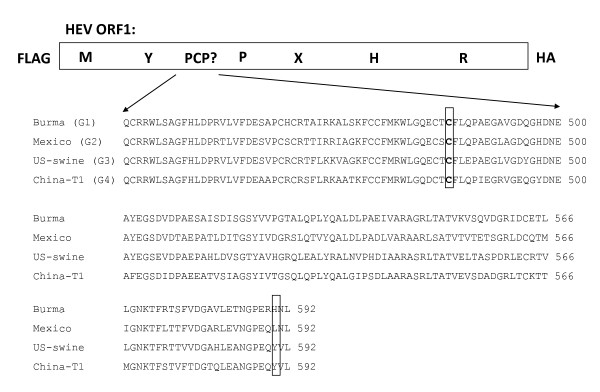
**Schematic diagram of expressed HEV ORF1 and multiple sequence alignment of the putative PCP domain in genotypes 1-4. **ORF1 of HEV is shown schematically as a box containing a number of motifs identified by computer-assisted homology searching [2]. The motifs are: methyl/guanylyl transferase (M), Y domain (unknown function), papain-like cysteine protease (PCP?, the presence of which is tested in this study), proline rich region (P), X domain (poly ADP ribose phosphatase), helicase (H) and RNA-dependent RNA polymerase domain (R). For expression, ORF1 was amplified from pTM1HEV, a plasmid used to express ORF1 of the Burma strain of HEV (genotype 1) in our earlier study [1], and pSHEV3, an infectious cDNA clone of the swine US strain of HEV (genotype 3)[14](obtained from X.J. Meng), by PCR using primers that added a FLAG epitope at the N-terminus and an HA-epitope at the C-terminus of the ORF. The multiple sequence alignment consists of the putative PCP of representative members of the four HEV genotypes (Genotype 1, Burma strain M73218; Genotype 2, Mexico strain M74506; Genotype 3, swine US strain AF082843; Genotype 4, China T1 strain AJ272108). An alignment of 135 HEV ORF1 from HEV genomic sequences available on GenBank revealed that the putative cysteine catalytic residue (C483, boxed) is conserved while the putative catalytic histidine residue (H590, boxed) is present in genotype 1 sequences, but is not conserved in the other genotypes. It should be noted that the catalytic cysteine and histidine residues of the PCP in the NS-ORF of rubella virus are conserved in all eight rubella virus genotypes (Yumei Zhou, unpublished data).

Hepatitis E virus (HEV) is the sole member of the genus *Hepevirus *belonging to the family *Hepeviridae *[reviewed in [3]]. HEV is a non-enveloped, plus-sense, single-stranded RNA virus whose genome is approximately 7.2 kb in length and consists of three open reading frames (ORFs): the 5' proximal ORF1 of ~5100 nt which encodes the viral replicase components; the 3' proximal ORF2 of ~2000 nt which encodes the capsid protein; and ORF3 of ~400 nt, which encodes a phosphoprotein of unknown function. ORF2 and ORF3 overlap and are translated from a single subgenomic RNA species. Computer-assisted sequence alignment of the deduced translation product of ORF1 with replicase proteins of other animal and plant plus-strand RNA viruses led to the identification of several common motifs, including putative methyl/guanylyl-transferase, papain-like cysteine protease (PCP), poly ADP ribose phosphatase, helicase and RNA-dependent-RNA-polymerase (RDRP) domains [2](see Figure 1). Of these, activity of the methyl/guanylyl transferase, the helicase, and the RDRP domains have been experimentally demonstrated [4-7].

Proteolytic processing of replicase polyprotein precursors into mature protein products is a common mechanism among plus-sense RNA viruses and has been demonstrated for all plus-sense RNA viruses of animals. HEV is most closely related to the two genera of the Togaviridae family, the alphavirus genus and the rubivirus genus. The replicase precursors of the viruses in these genera are processed into four and two mature proteins, respectively [8,9]. For both of these genera, it has been shown that processing regulates the synthesis of plus- and minus-strand RNA synthesis [10,11]. Because the enzyme responsible for proteolytic processing resides within the precursor, authentic processing occurs when the replicase precursor is expressed. Processing of the primary HEV ORF1 translation product, which has a putative MW of ~185 kDa, has not been resolved. No processing was detected when ORF1 was translated *in vitro *and the main product following expression of ORF1 in a number of mammalian cell lines was the ~185 kDa uncleaved species, however putative processing products were detected [1,12]. Expression of ORF1 by baculovirus in insect cells yielded a putative total of eight cleavages [13].

With respect to our earlier study, vaccinia virus-based expression of ORF1 derived from the human Burma strain of HEV (genotype 1) revealed the presence of putative N- and C-terminal products of 78 kDa and 107 kDa as well as the 185 kDa uncleaved species [1]. However, mutation of the putative catalytic cysteine (C483) of the predicted protease catalytic site within ORF1 did not eliminate these products and thus it could not be ruled out this processing might be due to the expression system employed. Therefore, to begin this follow up study, we tried expression of the Burma strain ORF1 using a protease-free vector, namely a plasmid vector, VR1012 (Vical, Inc., San Diego, CA), in which expression is driven by the human cytomegalovirus (CMV) immediate early promoter. In this construct, ORF1 was tagged at its N- and C-termini with FLAG and HA epitopes, respectively. This time, the 185 kDa uncleaved product was again the predominant species, however a putative N- terminal product with an apparent molecular weight of 115 kDa was detected with no corresponding putative C-terminal product (data not shown).

**Figure 2:**
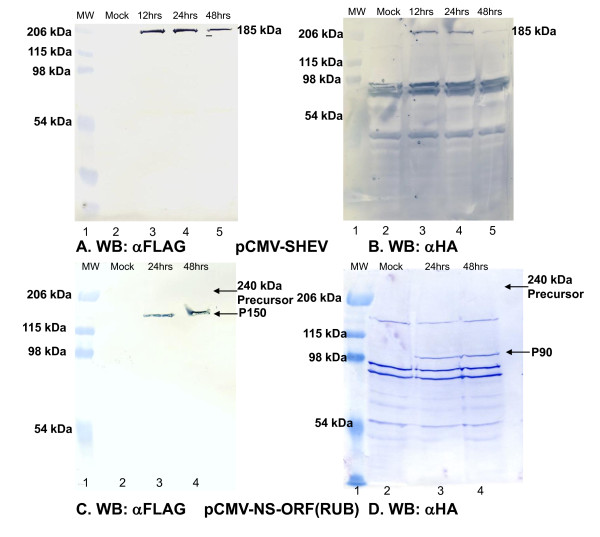
**Expression of US swine HEV ORF1 and rubella virus non-structural ORF. **293T cells were transfected with 5 μg of pCMV-SHEV DNA using Lipofectamine-2000 (as recommended by manufacturer's protocol) and harvested 12, 24, and 48 hours post-transfection. Mock transfected cells were processed similarly as a negative control. The cells were washed twice with PBS and lysed with 1X NP-40 buffer (1% NP-40, 150 mM Nacl, 50 mM Tris-HCl at pH 7.4 and 2 mM EDTA) in the presence of 1X complete mini, EDTA-free protease inhibitors (Roche). The lysates were resolved on 8% SDS-PAGE gels followed by transfer of the contents to nitrocellulose membranes and immunoblotted with for 1 h with anti-FLAG antibody-peroxidase conjugate from Sigma (Panel A) or anti-HA antibody- peroxidase conjugate from Roche (Panel B). Membranes were washed 5 times with 0.05% T-TBS (0.5 ml Tween-20 in 1 L 1X TBS [20 mM Tris-HCl at pH 7.5 and 175 mM NaCl]). The peroxidase was detected using BM Blue POD substrate (Roche). Lane1: Broad range molecular weight standard marker (M_r_'s in kDa given on left margin); Lane2: Mock transfected cells; Lanes 3-5: pCMV-SHEV-transfected cells harvested at 12, 24, and 48 hrs post-transfection (the 185 kDa ORF1 translation product is denoted on the right margin). As a control, the nonstructural protein ORF (NS-ORF) of rubella virus was amplified from Robo502, an infectious cDNA clone (18), by PCR using primers that added a FLAG epitope at the N-terminus and an HA-epitope at the C-terminus of the ORF and cloned into VR1012 plasmid vector; the resulting construct was termed pCMV-NS-ORF. 293T cells were transfected with 5 μg of pCMV-NS-ORF DNA using Lipofectamine-2000 (as recommended by manufacturer's protocol) and harvested 24 and 48 hours post-transfection. Mock transfected cells were processed similarly as a negative control. Western blotting and probing of lysates was done as described in the legend to Figure 2. Lane 1: Broad range molecular weight standard marker (M_r_'s in kDa given on left margin); Lane 2: Mock transfected cells; Lanes 3-4: pCMV-NS-ORF-transfected cells harvested at 24 and 48 hrs post-transfection. The 240 kDa precursor and the P150 (N-terminal) and P90 (C-terminal) products are denoted in the right margin.

It was not clear why putative processing of ORF1 of the Burma strain differed between the two expression systems. However an infectious cDNA clone was never assembled from the Burma strain of HEV and therefore to guard against artifacts due to potential mutations in the ORF1 construct assembled from this strain, we expressed ORF1 from pSHEV3, a cDNA clone of the US swine strain (genotype 3) that was shown to be infectious [14]. ORF1 from pSHEV3 was introduced into the plasmid vector with N-terminal FLAG and C-terminal HA epitope tags, resulting in a construct termed pCMV-SHEV. As shown in Figure 2A and 2B, expression of the 185 kDa product was detected with both anti-FLAG and anti-HA antibody but no other ORF1-specific products were apparent (background bands of ~95, 90, and 55 kDa were detected by the anti-HA antibody).

As a positive control to determine if processing of a virus nonstructural replicase protein precursor could be detected using this expression system, the rubella virus nonstructural protein ORF (which corresponds to ORF1 of HEV) from an infectious cDNA clone of rubella virus [15] was also provided with N-terminal FLAG and C-terminal HA tags and cloned into the VR1012 vector, yielding a construct termed pCMV-NS-ORF. As shown in Figure 2C and 2D, when this construct was expressed, the N-terminal P150 protein was detected with anti-FLAG antibody and the C-terminal P90 protein was detected with anti-HA antibody. No 240 kDa precursor product was detected with either antibody, indicating that processing was complete.

Taken together, there is no consistent evidence that the HEV ORF1 primary translation product undergoes proteolytic processing. Expressed ORF1 from an Indian strain of HEV (genotype 1) and US swine strain (genotype 3) exhibited no processing while potential processing products of the ORF1 of the human Burma strain (genotype 1) were not consistent between different expression systems and therefore are likely artifactual [1,12]. ORF1 of the Indian strain underwent a complex processing scheme when in expressed by baculovirus in insect cells [13], but it must be considered that insects are not the natural hosts for HEV. HEV *in vitro *genomic and replicon (constructs in which the structural proteins are replaced with reporter genes) RNA transcripts that are infectious in both cell culture and animals have been available for several years [14,16-20]. Despite the potential of these systems to resolve whether ORF1 processing occurs during replication, convincing evidence one way or the other has yet to be reported. Thus, HEV potentially is unique among plus-sense RNA viruses of animals in the feature of lacking proteolytic processing of its replicase precursor. In this regard, the catalytic residues of the putative PCP that was postulated to mediate processing of ORF1 were predicted on the basis of the original HEV sequence (genotype 1 Burma strain)[2]. Since that time, numerous additional sequences of HEV have been reported and four genotypes have been distinguished. A search of GenBank yielded 135 complete genomic sequences of HEV and alignment of ORF1 from these sequences revealed that while the putative catalytic cysteine residue (C483) of the predicted PCP is conserved across these sequences, the putative catalytic histidine residue is not (Figure 1). In fact the residue at position 590 of ORF1 is genotype specific: H in genotype 1, L in genotype 2, and predominantly Y in genotypes 3 and 4. Additionally, in the recently described avian HEV the region of ORF1 containing the predicted PCP is not present, consistent with lack of processing of ORF1 [21].

## Competing interests

The authors declare that they have no competing interests.

## Authors' contributions

SS carried out the research and participated in drafting of the manuscript. YMZ conducted the ORF1 alignments and participated in drafting of the manuscript. TKF, as senior author, advised SS on the research, participated in drafting of the manuscript, and serves as corresponding author. All three authors have read and approved the final manuscript.

## Author information

The research described in this report was conducted by SS as part of her PhD dissertation. SS is currently a Postdoctoral Fellow in the Department of Pathology, Emory University School of Medicine.
